# A Review on Metallic Alloys Fabrication Using Elemental Powder Blends by Laser Powder Directed Energy Deposition Process

**DOI:** 10.3390/ma13163562

**Published:** 2020-08-12

**Authors:** Yitao Chen, Xinchang Zhang, Mohammad Masud Parvez, Frank Liou

**Affiliations:** Department of Mechanical and Aerospace Engineering, Missouri University of Science and Technology, Rolla, MO 65401, USA; yc4gc@mst.edu (Y.C.); mphf2@mst.edu (M.M.P.); liou@mst.edu (F.L.)

**Keywords:** metal additive manufacturing, directed energy deposition, alloy design, elemental powder mixture, advanced materials, composition control

## Abstract

The laser powder directed energy deposition process is a metal additive manufacturing technique, which can fabricate metal parts with high geometric and material flexibility. The unique feature of in-situ powder feeding makes it possible to customize the elemental composition using elemental powder mixture during the fabrication process. Thus, it can be potentially applied to synthesize industrial alloys with low cost, modify alloys with different powder mixtures, and design novel alloys with location-dependent properties using elemental powder blends as feedstocks. This paper provides an overview of using a laser powder directed energy deposition method to fabricate various types of alloys by feeding elemental powder blends. At first, the advantage of laser powder directed energy deposition in manufacturing metal alloys is described in detail. Then, the state-of-the-art research and development in alloys fabricated by laser powder directed energy deposition through a mix of elemental powders in multiple categories is reviewed. Finally, critical technical challenges, mainly in composition control are discussed for future development.

## 1. Introduction

**Figure 1:**
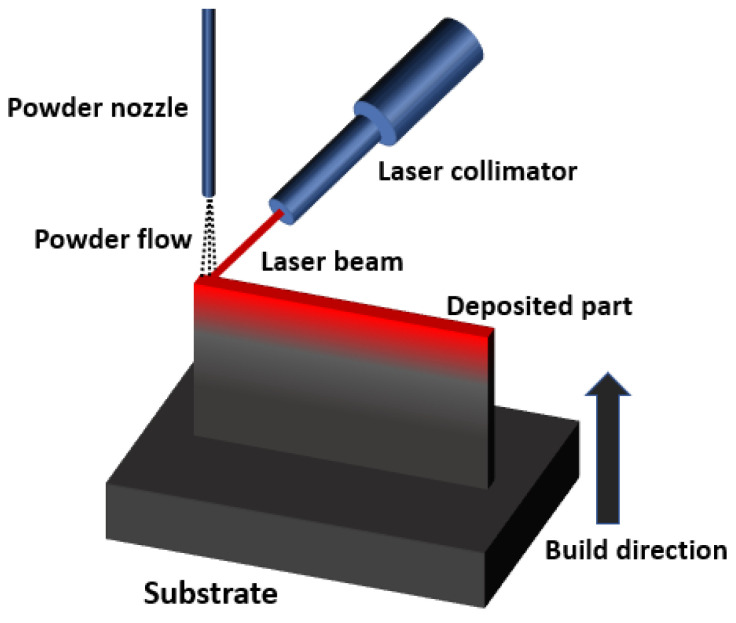
Schematic illustration of DED to build a 3D part.

Additive manufacturing (AM) is a novel manufacturing technique that can fabricate a wide range of materials and complex structures. The definition given in the American Society for Testing and Materials (ASTM) states that: AM is “The process of joining materials to make objects from 3D model data, usually layer upon layer, as opposed to subtractive manufacturing methodologies [1]”. Due to its layer-based additive nature that is different from subtractive manufacturing, AM created a paradigm shift in the manufacturing industry [2]. AM has lots of advantages compared to traditional subtractive manufacturing. For example, AM can directly produce complex 3D parts without much tooling and assembly. It is also much more material-saving than conventional manufacturing since conventional manufacturing mainly uses the subtractive method to remove materials to reach the desired geometry [3]. Thus, AM has become more essential in the manufacturing industry. As metals and their alloys are of great importance in human life, efforts have been paid on the research and development of AM of metals and alloys [4]. Based on the mechanism and material, the AM process has been classified into seven categories [5]. There are four major categories associated with metal additive manufacturing, which are powder bed fusion (PBF), directed energy deposition (DED), binder jetting (BJ), and laminated object manufacturing (LOM). Among them, PBF and DED are more commercialized [5]. According to ASTM F3187-16 standard guide for DED technique [6], the DED process applies an energy source to fuse feedstock metallic materials by melting during deposition. Metallic materials in powder or wire form are fed into the melt pool and solidify into a 2D solid layer. The tool path is guided by path planning to fill every layer, and the successive layers will be built until a 3D part is achieved [6]. The laser powder DED process applies laser as the energy source and metal powder as the raw material feedstock. In this paper, we focus on the laser powder DED, and for convenience, here we use DED to represent the laser powder DED process. Using computer-aided design (CAD) tools, a 3D model of a part can be created, and the slicing algorithm can be used to slice the 3D model into many 2D layers. Figure 1 shows the schematic diagram of the DED process using powder as feedstock. During the DED process, a laser beam is applied to create a melt pool, and metal powders are carried and blown into the melt pool by the inert gas. After the laser moves away, melted powders will join and cool down to form a solid layer. The laser travels according to the toolpath for each layer. By repeating this procedure for each layer, a 3D part can be constructed [7].

During the past decade, typical metal alloys fabricated by DED has been studied. These alloys include austenitic stainless steels (304/304L [8,9] and 316/316L [10,11]), precipitation hardening stainless steels (17-4 PH [12]), nickel-based superalloys (Inconel 718 [13,14] and Inconel 625 [15,16]), titanium alloys (Ti-6Al-4V [17,18,19,20]), etc. DED and PBF have their own advantages according to their special features. For DED, it is able to build parts on a non-flat surface while PBF usually needs a horizontal area for powder spreading. Thus, modification of the curved surface and repairing of damaged parts are also possible using the DED process [21,22,23]. The part remanufacturing can be collaborated with reverse engineering to repair damaged parts by constructing a damaged profile and determine the laser scanning strategy, which will be of great significance in saving cost on tooling [24,25]. As an in-situ powder feeding process, it has the potential to fabricate parts without an enclosed chamber [26]. Therefore, the volume of building parts can be much larger than the PBF process. One more important feature is because of the in-situ powder feeding process, DED can flexibly create different material compositions from layer to layer by mixing different powders; however, this is difficult to be realized in PBF. Therefore, much more metallic alloys with various compositions can be potentially directly created by taking this advantage of DED. This novel aspect of DED will be the main topic to review in this article.

Metal powders are the commonly used raw materials and feedstocks in the DED process. Most of the powders used in DED mentioned in the former paragraphs are pre-alloyed powders, which indicate that each powder particle is designed with the prescribed composition. Since the composition is identical in each particle, the composition of the as-fabricated 3D part made by DED is usually close to constant. However, the cost of producing pre-alloyed powders are high. Therefore, similar to using cost-effective elemental powders in powder metallurgy [27,28,29], the idea of mixing elemental powders into the desired composition to synthesize alloys is also arisen in the area of AM, especially for DED. The cost of alloy powder manufacturing can be reduced by using elemental powders. Also, as each pre-alloyed powder particle has a constant elemental composition, the possibility of fabricating various types of alloys using DED is limited. If the pre-alloyed powders are replaced by elemental powder blends, it is possible to fabricate more alloys with a pre-designed elemental weight percentage or atomic percentage. This replacement can potentially make a great contribution to the development of novel alloys through the thorough investigation of different alloy systems. In addition, with the evolvement of highly automated DED manufacturing systems, the weight composition of multiple elemental powders can be changed during the manufacturing process by in-situ control of the feeding rate. Then, a variety of elemental compositions can coexist within a single part, which can be more functional than homogeneous alloys.

Although a few works have been done using DED to fabricate various alloys, as a relatively new method in DED, a comprehensive overview of the research progress of DED using elemental powder blends has not been done. Thus, an overview of the elemental powder-based DED process can provide new knowledge systems for the metal AM area and potentially develop new alloys, which can significantly widen the application of metal AM in the next generation of manufacturing fields. This review paper will summarize the current research progress in different types of applications via DED and discuss the major technical challenges and issues that remained in this area in order to provide guidelines for future studies.

## 2. Current Status of DED Using Elemental Powder

The typically reported research works in DED using elemental powders can be generally classified into two categories, which are listed and elaborated in Section 2.1 and Section 2.2, respectively. Many types of industrial alloys can be potentially fabricated by mixing the specified composition of elemental powders. Also, conventional alloys can be flexibly modified by mixing elemental powders with other compositions to get an in-depth understanding of how a certain element affects the final properties. The study of the effect of specific elements on the as-fabricated parts will be more direct. With the flexible change in compositions, various types of alloys with a gradual shift in alloy compositions can be joined and form functionally graded materials (FGMs). Using DED and elemental powder blend method can investigate FGMs that are difficult to realize using conventional manufacturing. Novel alloys, especially high entropy alloys (HEAs), which need more types of elements, can be fabricated and designed flexibly by the DED process using elemental powders. Section 3 mainly covers the controlling of the deposition in multiple aspects. The outlook is discussed in Section 4, while the conclusion is summarized in Section 5.

### 2.1. Industrial Alloys and Intermetallics

There is a wide variety of alloys that are prevalent in the industry due to their excellent mechanical properties. However, due to the high manufacturing cost, they are mostly seen in specific areas. For instance, Ti-6Al-4V is an excellent industrial alloy with high specific strength and corrosion resistance [30]. However, the cost is high for certain industrial areas such as automobile and transportation [31]. Thus, reducing the manufacturing cost of Ti-6Al-4V is essential to expand the applications. Near net shape processing, such as powder metallurgy, was reported as a cost-effective approach to develop and expand the use of titanium alloys [32]. In the area of powder metallurgy, the elemental powder blend is applied to form titanium alloys and avoid the high cost of pre-alloyed powders [32]. Based on the elemental powder method applied in powder metallurgy, blending elemental powder to fabricate alloys has also become a potential method in powder-based metal AM, such as DED, to reduce the manufacturing cost and open new perspective research and industrial fields. Until now, several attempts were made in synthesizing Ti-6Al-4V by a mixture of Ti, Al, and V pure powders via DED. Differences were found between Ti-6Al-4V fabricated by DED using elemental powder blends and conventional Ti-6Al-4V. Manufacturing issues were also identified. More investigation of properties and performances of DED-processed Ti-6Al-4V using elemental powder blends are needed.

Hua et al., Yan et al., and Chen et al. [33,34,35,36] mixed Ti, Al, and V elemental powders to fabricate Ti-6Al-4V, which proved the feasibility of making industrial alloys like Ti-6Al-4V using elemental powders in a cost-effective approach. Apart from Ti-6Al-4V, Clayton [37] applied pure Fe, Cr, and Ni powders to make Fe-based alloys similar to stainless steels such as SS316 and SS430. It was found that the Fe-based alloy Fe-17Cr-12Ni made by elemental powder mixture got similar microstructure and mechanical properties with SS316. On the other hand, properties of the alloy Fe-17Ni fabricated by elemental powder mixture was not comparable with the conventional pre-alloyed SS430. More experimental investigations are needed to reveal the attributes of the difference in properties. Similarly, a number of other Fe-, Ti-, and Ni-based alloys in Fe-Cr-Ni and Ti-Al-V alloy systems can be potentially fabricated by mixing elemental powders with certain compositions. It is significantly beneficial to the alloy manufacturing industry that using only a small stock of Fe, Cr, Ni, Ti, Al, and V powders can generate a large number of alloy combinations.

Some types of intermetallic compounds possess excellent mechanical properties that could be widely used in various industries. Thanks to the manufacturing flexibility of the DED process, many hard-to-machine intermetallic compounds can now be fabricated by new methods. As a wide variety of intermetallic compounds only consist of two metal elements, mixing elemental powder can be a convenient way to synthesize those compounds. A couple of intermetallics are attractive for their high wear and corrosion resistance. For instance, Fe-Al intermetallics possess excellent wear, corrosion, and oxidation resistance. It can also function in a high-temperature environment. Conventionally, Fe-Al intermetallics are manufactured by sintering blended elemental powders. However, this process causes high energy consumption and cost [38]. Therefore, the DED process was also introduced for the fabrication of Fe-Al intermetallic [38]. Pęska et al. [39] applied elemental Fe and Al powder to synthesize Fe-Al intermetallics by DED. The hardness of the as-fabricated samples was very similar to the classically built Fe-Al intermetallics.

**Figure 2:**
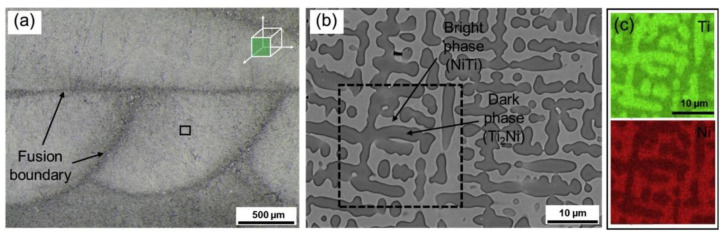
(**a**) Microstructure and fusion boundary of NiTi alloy fabricated by DED using elemental powder blends. (**b**) SEM image of the NiTi alloy with NiTi phase and Ti_2_Ni phase. (**c**) EDS mapping of Ti and Ni [49]. (Reproduced with permission from Elsevier).

NiTi is a special intermetallic compound with unique shape memory effects and superelasticity. It is also biocompatible and corrosion resistive [40]. Thus, it is widely used in structures with shape-changing effect and biomedical implants. Machining is difficult to process NiTi. To reduce the machining procedure, attentions are paid on near-net shaping processes, especially AM. Attempts were made by using the DED process to fabricate NiTi and pre-alloyed powders were applied in [41,42,43]. As pre-alloyed NiTi powder is expensive, blending Ni and Ti elemental powder is an alternative way to in-situ synthesize Ni-Ti alloys and with a variety of composition design. Halani et al. [44] applied the DED process to fabricate NiTi using elemental powder. Different compositions such as Ni55Ti45 and Ni50Ti50 in atomic percentage were attempted for the deposition process. Similarly, Shiva et al. [45] studied the difference among premixed compositions of Ni45Ti55, Ni50Ti50, and Ni55Ti45. Bimber et al. [46,47,48] conducted works in fabricating NiTi by DED with elemental powder blends. They built large volume structures to find out the difference in the secondary phase, compressive properties, and martensitic transformation temperature regarding the spatial locations. Other works include comparative studies among DED, SLM, and EBM by Wang et al. [49]. Figure 2 shows the microstructure of NiTi alloy fabricated by DED in [49]. Different issues were identified in three metal AM processing methods, which will be discussed in the later sections.

Other types of intermetallics for surface strengthening using elemental powder mixture for DED were also studied. Most of the works focus on the surface strengthening of steel and titanium alloy to improve wear and corrosion resistance by synthesizing intermetallics on the surface. Yu et al. [50] applied elemental Al and Ni powder to synthesize pure Ni_3_Al intermetallic compound coating on 1Cr18Ni9Ti stainless steel. Effects of laser energy density on tribological behavior and crystallographic orientation were studied. Wang et al. [51,52,53,54] have fabricated various types of intermetallics for surface coating applications using laser cladding. Those works include coating TiCo/Ti_2_Co on titanium alloy [51], coating Ti_5_Si_3_/NiTi_2_ on titanium alloy [52], and coating Cr_3_Si on austenitic stainless steel 1Cr18Ni9Ti by using Cr-Si-Ni elemental powder as the precursor material [53]. Si was also blended with metal powders to provide intermetallics and Ni_2_Si/NiSi on 0.2% C carbon steel [54]. Zhong et al. [55] applied the DED process to clad WC/Ni hardfacing alloy on 40Cr steel by using W, C, and Ni elemental powder. The in-situ reaction produced WC hard phase in the Ni matrix for surface wear resistance improvement. Different dissolution behaviors were observed in W and C/Ni powder within the melt pool at different locations in the melt pool. The dissolution situation of W, C, and N depends highly on the local temperature distribution and reheating, which is related to laser deposition parameters and deposition strategies.

### 2.2. Develop Advanced Alloys

Due to the advantage of customization and small-batch manufacturing, the DED process with blended elemental powders is also a powerful tool for developing novel alloys and inventing innovative materials [33]. Changes in structure and property of adding different types and quantities of elements in an alloy system can be quickly observed by a small volume deposition, rather than making a large structure using conventional methods. By adding more alloying elements, the fabrication of advanced alloys such as FGMs, HEAs, and metallic glass can also be realized. Therefore, using elemental powder will then create many more probabilities in the field of alloy design in a cost-effective way.

#### 2.2.1. Alloy Modification

Modifying alloys can be flexible by using elemental powders in DED, as the composition can be customized by varying parts of the elements or all elements. This method may either solve the difficulty in processing certain alloys or study the element effects in alloy systems using DED. This advantage can benefit the development of numerous binary and ternary alloy systems, including, but not limited to Cu-Ni, Ti-Nb, Fe-W, Ti-Al-Mo, etc. [56,57,58,59] and the development of new alloys. Cu and Ni are completely soluble, which attracts industrial interests in making Cu-Ni alloys with both high thermal conductivity from Cu and high mechanical properties from Ni. Karnati et al. [56] mixed elemental Cu and near-pure Ni powders in different composition levels, and they all produced solid solutions of Cu-Ni alloys. Li et al. [57] deposited 80W-20Fe using elemental W and Fe powders, which indicates DED is an effective and novel method to process W based alloys. Fallah et al. [58] deposited 55 wt.% Ti/45 wt.% Nb in elemental powder blend on the Ti-6Al-4V substrate to create a compositionally modified surface layer. Then the biocompatibility of Ti-Nb alloy at the surface can be utilized, and the cost can be reduced by avoiding manufacturing the entire part using Ti-Nb alloy. Zhang et al. [59] deposited a series of Ti-2Al-yMo (y = 2, 5, 7, 9, 12) to study the Ti-Al-X system and develop innovative alloys via DED.

#### 2.2.2. FGMs

**Figure 3:**
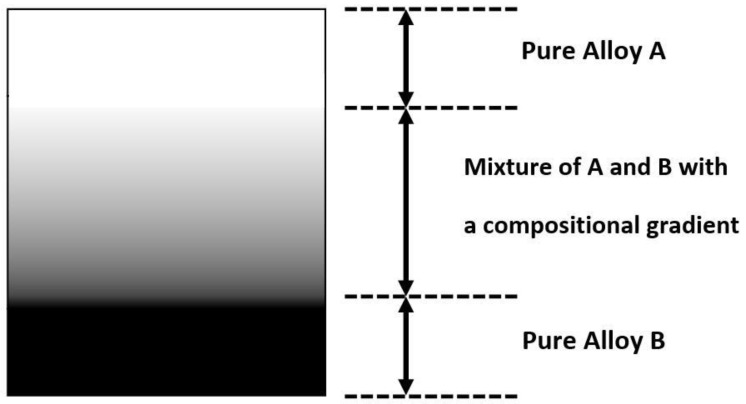
FGM joined by pure alloy A and pure alloy B with a compositional gradient.

**Figure 4:**
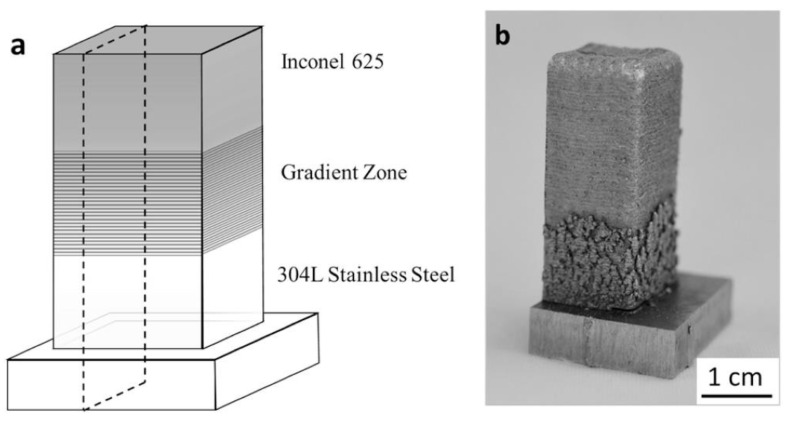
(**a**) Schematic of Inconel 625/304L stainless steel FGM. (**b**) Image of an Inconel 625/304L stainless steel FGM sample fabricated by DED [61]. (Reproduced with permission from Elsevier).

The elemental powder mixture can be used to modify alloys using different compositions of elemental powders. The DED process is also a flexible layer-based AM technique, which can produce different compositions at different locations. Then, within a certain binary or ternary alloy system, different compositions can be joined together by taking a proper usage of the DED process in a layer-wise fashion. More advanced metallic alloys can be designed for multifunctionality, and FGM is a good example. The concept of FGM originated from applying a graded composition between two materials with different properties to avoid delamination under extreme loading conditions at the interface [60]. Compared with selective laser melting (SLM), which is a laser-based metal additive manufacturing in the category of PBF, one important advantage for DED is the flexibility of in-situ control of the location-dependent chemical composition. The flexibility in composition control makes DED an excellent processing technique for fabricating FGMs [60]. DED can be fully utilized to deposit different compositions of elemental powder mixtures layer by layer without using complicated assembly processes in traditional manufacturing. One purpose of fabricating FGMs is to join two dissimilar pure alloys without a sharp interface [61,62,63,64]. This could be solved by adding compositionally graded interlayers between pure alloy A and pure alloy B. Figure 3 illustrates the concept of joining based FGM, and a real deposited sample from the literature [61] is shown in Figure 4, which joins Inconel 625 and 304L stainless steel via a compositional gradient.

**Figure 5:**
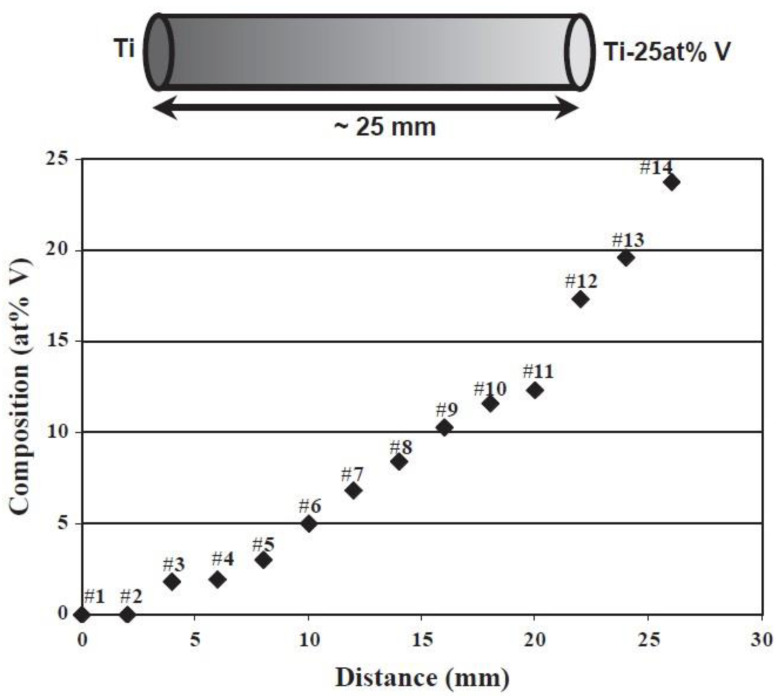
Schematic diagram of Ti-25 at.%V FGM alloy fabricated via elemental powder mixture and the compositional variation vs. distance [65]. (Reproduced with permission from Elsevier).

In addition, FGMs can be intentionally designed by combining different chemical compositions at different locations to fabricate multifunctional parts. This type of FGM has been done in using the DED method to fabricate binary or ternary alloy systems with a compositional gradient via elemental powder mixture. Banerjee et al. have made a decent amount of Ti-based binary alloys into FGMs using elemental powders, including Ti-V [65,66], Ti-Mo [66], and Ti-Ta [67]. Titanium can form α/β alloy systems with many other metal elements. As seen in Figure 5, a Ti/V FGM was fabricated by mixing graded Ti/V composition and the resultant composition gradient varies from 100% Ti on the left to 75 at.% Ti/25 at.% V on the right side. It is of great interest to apply elemental powder to study graded titanium alloys and identify their process-structure-property relations since many Ti-based binary alloys have not been widely considered in the AM category. Elemental powder mixture will be convenient to check the effect of alloying elements on microstructure, grain size, and mechanical properties. Those works focused on the relationship among composition, microstructure, and mechanical properties of Ti-based binary alloys with a composition change along the graded direction. Here the research in DED-processed FGMs also helps to establish the process-structure-property relationship of these promising alloy systems. Ti-Cr [68], Ti-Al [69], Ti-W [70], and Ti-Mo [71] systems were also investigated. Details are tabulated in Table 1. W element is good for grain refinement, while Mo does not have a significant refining effect. They also studied the Ti-Al-V system by varying the composition of V using the elemental powder mixture of Ti-8Al-xV [72]. Thus, graded ternary alloys can also be fabricated using DED to systematically study the effect of variation of alloying elements.

**Figure 6:**
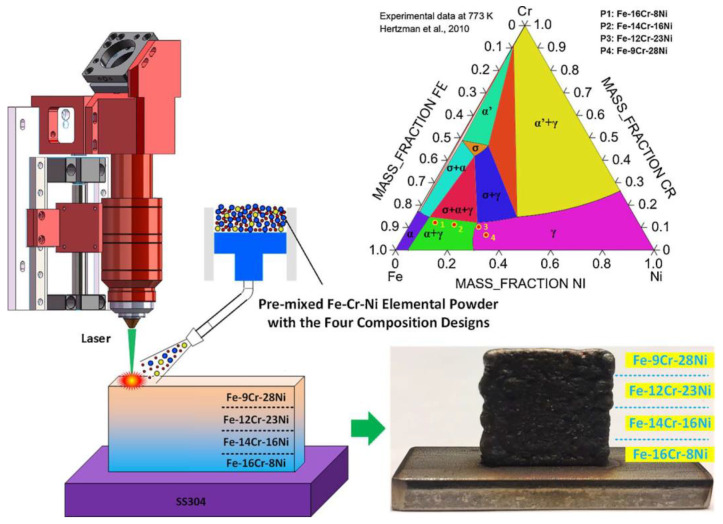
Fabricating Fe-Cr-Ni FGM by DED process using elemental Fe, Cr, and Ni powders [74]. (Reproduced with permission from Elsevier).

Karnati et al. [73] mixed elemental Cu and Ni powders to create Cu-Ni FGMs based on the complete solubility in Cu-Ni binary system. After the previous investigation of mixing Cu and Ni in different compositions, the different compositions were then combined and fabricated into Cu/Ni FGM. Li et al. [74,75] fabricated a new graded Fe-Cr-Ni alloy using Fe, Cr, and Ni elemental powders with a gradient in Cr and Ni composition, as shown in Figure 6. Thus, on the Cr-rich side, the graded system can exhibit excellent behaviors in corrosion resistance. On the Ni-rich side, the system possesses high plasticity owing to the large composition of austenite.

#### 2.2.3. Magnetic Materials and Metallic Glass

Apart from common structural alloys, new types of alloy systems with special functions were also studied using elemental powder blend. Conteri et al. [76] studied a novel magnetic alloy Fe73.5Si13.5B9Nb3Cu1. Based on this study, Borkar et al. [77] synthesized a more complex magnetic alloy with a gradually changing Si/B ratio. Thus, this new design is also known as a functionally graded Fe-Si-B-Cu-Nb alloy with magnetic properties. Amorphous (or glassy) metals can be fabricated using this technique. Manna et al. [78] deposited 94Fe4B2C, 75Fe15B10Si, and 78Fe10BC9Si2Al1C by mixing glass-forming elemental powders on a substrate made by carbon steel. While in Hou et al.’s work [79], the Fe-based Fe-Cr-Mo-Co-C-B amorphous alloy was produced according to the weight percentage of Fe45.8Mo24.2Cr14.7Co7.8C3.2B4.3. The amorphous phase occupied 52.8% of the entire volume. The resulted hardness of the deposition has a maximum of 1200 HV0.5, which shows a significant improvement compared to the substrate that is 200 HV0.5.

#### 2.2.4. HEAs

HEA was also proved to be feasible to be fabricated by DED using elemental powders. HEAs are known to possess high hardness, excellent high-temperature strength, corrosion resistance, and wear resistance due to the unique multiprincipal element composition [80]. It is promising for fabricating coating materials on engineering parts for wear and oxidation resistance. As the flexible mixture of powders from at least five principal elements, different atomic ratios can be varied to study the element effect on the as-fabricated HEAs, such as Al_x_CrCuFeNi [81], where x varies while the atomic percentage of all five elements are between 5 at.% and 35 at.%.

Coating HEAs on conventional structural alloys are highly attractive due to the potential high hardness of HEAs. Cui et al. [82] applied DED to coat AlCoCrFeNi on AISI SS316 using the elemental powders. An intermediate CoFe_2_Ni layer was applied between the AlCoCrFeNi HEA coating and the SS316 substrate to avoid cracking. The intermediate CoFe_2_Ni was also synthesized by elemental powder, which has the purpose of providing an average coefficient of thermal expansion (CTE) that does not differ greatly from the SS316 substrate and the HEA coating. Chao et al. [83] applied DED to coat Al_x_CoCrFeNi on a 253MA steel substrate, where the value of the Al mole fraction x was taken as 0.3, 0.6, and 0.85. Elemental powders were utilized, and the composition change of Al can be adjusted. The effect of the Al mole fraction on the crystal structure was revealed by material characterization. Chen et al. [84] varied two types of elements (Al and Cu) to study the influence on the structure and properties of Al_x_CoFeNiCu_1-x_, where x = 0.25, 0.5, and 0.75. It was found that crystal structure and hardness varied significantly from 0.25 Al/0.75 Cu to 0.75 Al/0.25 Cu. In another work, the hardness of Al alloy was improved by depositing Al_0.5_FeCu_0.7_NiCoCr HEA coating [85]. The average value of hardness reached about eight times higher than the Al alloy substrate.

In addition, HEAs that possess high erosion and oxidation resistance can be synthesized by elemental powder mixture. Siddiqui et al. [86] coated Al_x_Cu_0.5_FeNiTi HEA on Al alloy AA1050 by elemental powder blend for erosion resistance. It was stated that the erosion rate was decreased mainly due to the improved microhardness of tough grains formed in HEA. The HEA coating using elemental powder is also studied for the potentially high-temperature oxidation resistance. Huang et al. [87] studied that depositing TiVCrAlSi on Ti-6Al-4V can improve the oxidation resistance of Ti-6Al-4V at 800 °C.

As high temperature fields can be created by the high-power laser beam, some works that focus on combining a series of metal elements with high melting points to produce refractory HEAs were also carried out. Dobbelstein et al. [88] produced TiZrNbHfTa from elemental powder blends for the first time. The mixing was homogeneous, and a high hardness was achieved. The effect of the mole fraction of one specific element was also studied. The W_x_NbMoTa HEA with the composition change in W was fabricated by Li et al. [89]. The mole fraction x was taken as 0, 0.16, 0.33, and 0.53. It was found the microhardness increases with the increase in W. Based on the flexibility in modifying the mole fraction, different HEAs with different mole fractions can also be fabricated together to make FGMs, which then becomes compositionally graded HEAs. By taking advantage of FGM, Dobbelstein et al. [90] also fabricated compositionally graded TiZrNbTa refractory HEAs using elemental powder blends. Gwalani et al. [91] deposited AlCrFeMoV_x_ compositionally graded HEA, where the mole fraction of V varies from 0 to 1. The lattice parameter decreased, and an improvement was found on hardness with the increase in V content. DED with elemental powders was regarded as a high-throughput method to study the composition-microstructure-hardness relationship of novel HEAs.

## 3. Deposition Control

In Section 2, major types of alloys which have been fabricated by elemental powder based DED technique are introduced and discussed. The classification and examples with the reference are tabulated in Table 2. Using elemental powders to fabricate various types of alloys using DED is now very promising. However, as a novel technique, there are still a lot of unsolved issues beyond feasibility studies to popularize this concept. Whether the composition of the deposited part matches up with the originally designed composition is a big issue for this technique. Also, the final deposition is expected to be homogeneous. As the entire feedstock delivery and manufacturing system are highly complex, multiple key factors should be taken into consideration. The stability and repeatability are of great importance to promote this process into a new stage in various industries.

### 3.1. Enthalpy of Mixing

Mixing is important to maintain a good homogeneity during the deposition. To ensure a uniform mixing, the enthalpy of mixing of the system should be negative. Schwendner et al. [92] used Ti-Cr and Ti-Nb binary elemental powder blend to study the effect of mixing enthalpy on the homogeneity of mixing. The results showed that Ti-10%Cr alloy has a negative enthalpy of mixing and has a homogeneous intermixing result within the melt pool. While the Ti-10%Nb system has an inhomogeneous mixing and a slow cooling rate. The mixing of enthalpy can be a useful criterion to guide the design of chemical elements in the alloy. For some of the alloy systems, the constituent elements are in the positive enthalpy of mixing, such as Cu-Ni [56] and Cu-Fe, the adjustment of laser power to generate additional energy for mixture homogenization is needed. For example, Karnati et al. [56] applied pulse width modulated laser power to induce vibrations in the melt pool to avoid segregation during the deposition of the Cu-Ni system.

### 3.2. Powder Delivery

Although using the elemental powder blend is straightforward to understand, the accuracy of the chemical composition of the as-deposited part is a big challenge. The pre-designed chemical compositions or elemental composition should match well with the deposited part. Element deviations are often observed from most of the previously reported works. Among those works, few of them made an in-depth investigation in this aspect. In [56], blending Ni and Cu resulted in the part with a 4% error with respect to the pre-designed composition. In [82], the atomic percentage of Al in AlCoCrFeNi HEA was between 10 at.% and 15 at.%, while the pre-designed atomic percentage was 20 at.%. In some cases, fabricating the material system with high compositional accuracy is of great importance. A small composition error can cause a significant change in microstructure, phase, and mechanical properties. For example, a slight increase in the Mo composition of Ti-Mo alloy will result in a bimodal distribution of α lath in the β matrix [66]. The control of chemical composition needs to be solved in order to improve the manufacturing quality of the elemental DED process and push this method entirely in the industrial applications.

**Figure 7:**
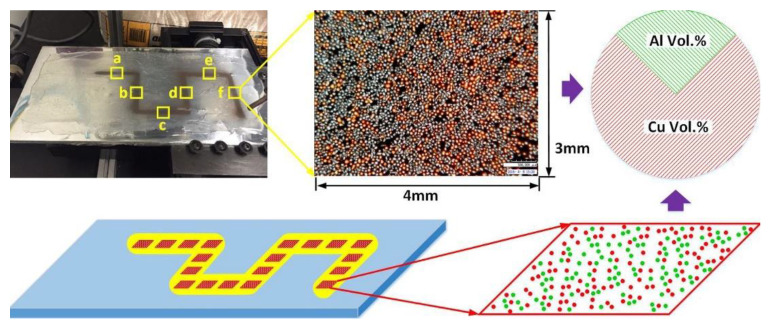
Investigation of powder segregation between Al powder and Cu powder by spraying powder mixture on a glue plate and calculating the volume ratio of Al powder and Cu powder at different locations [96]. (Reproduced with permission from Elsevier).

Powders are the commonly used feedstock material for the DED process. Most of the previous works applied the method of blown powder blend of pre-mixed elemental powders to deliver powders into the melt pool with the carrier gas. Since elemental powders made by different metals are mixed and delivered by carrier gas, the powder properties, and flow behavior are important to control. Collins et al. [93] mentioned that powder size and density could affect the actual composition of the as-deposited part in their work of fabricating Ti-Al-Mo and Ti-Al alloys. Li et al. systematically studied powder separation using both numerical and experimental methods [94,95,96]. As the powder mixture is delivered by the carrier gas, the velocity of each particle mainly depends on its shape, density, and size. Li performed studies in exploring the powder segregation in the blown powder DED process with pre-mixed spherical elemental powder as feedstocks, as seen in Figure 7. The larger the difference in density, the greater the segregation phenomenon will be. Cu and Al powders were applied for the experimental investigation of the flow behavior. In the investigation, it was found that significant segregation was observed in un-sieved powder mixtures without size adjustment. The segregation issue was resolved after the sieving process under the guidance of the density and size relation. The limitation of this model is it only works for spherical powder particles. For non-spherical powders, the flowability will be changed, and more studies are needed.

### 3.3. Capturing and Melting

When a real deposition is performed, a stream of powder is blown into the melt pool, and only part of the powder can be captured by the melt pool. The capture rate is also a factor to affect the chemical composition, as for different types of powders, the probability of falling into the melt pool also varies. Chen et al. [36] performed an experiment to investigate the powder capture rate of Ti, Al, and V at different size levels. For each metal powder, a constant capture rate can be determined, and the corresponding size level can be picked for the same capture rate. Another perspective for keeping a constant capture rate for different metal powders is to maintain a constant divergence angle to maintain a consistent composition before and after deposition. A mathematical model was worked out by Zhang et al. [97]. The constant divergence is a reflection of constant speed out of the nozzle. The optimized powder particle size of Ti, Al, and V used to maintain a constant divergence angle match nicely with Li’s work [94].

**Figure 8:**
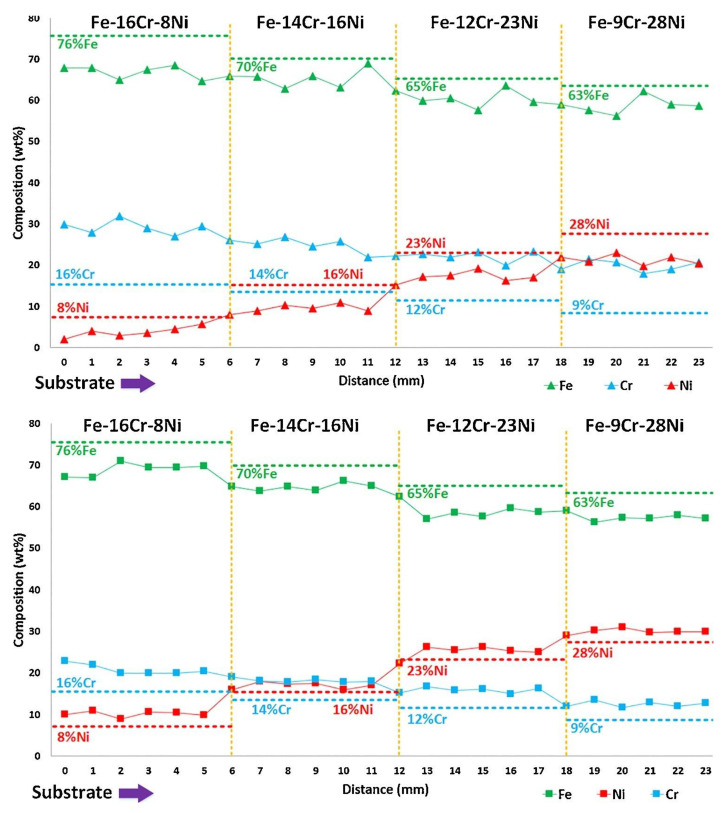
Element composition control of Fe-Cr-Ni FGM with un-sieved (the upper figure) and sieved (the lower figure) elemental powder mixture. The FGM fabricated by sieved powder mixture gives more accurate composition [74]. The percentage indicates wt.% in this figure. (Reproduced with permission from Elsevier).

The study of the flow behavior by Li et al. [96] revealed the relationship between powder properties and the avoidance of powder separation. However, it was limited in powder spray. The actual laser deposition was not performed to check the exact elemental composition of the as-deposited part. As when an actual deposition is performed, different metals may have different composition loss due to evaporation. The melt pool capture rate of powders can also be different. In the research [74,75] of the Fe-Cr-Ni FGM, Li et al. applied this method and found that the deposition works better when the powder size was sieved and controlled according to the physical properties of Fe, Cr, and Ni, as shown in Figure 8. Fe, Cr, and Ni have relatively similar density and melting point, the effect of evaporation and capture rate will be less obvious. For other combinations with larger differences in properties, such as Cu and Al differ greatly in density and melting temperature, further works should be done for this model extension.

**Figure 9:**
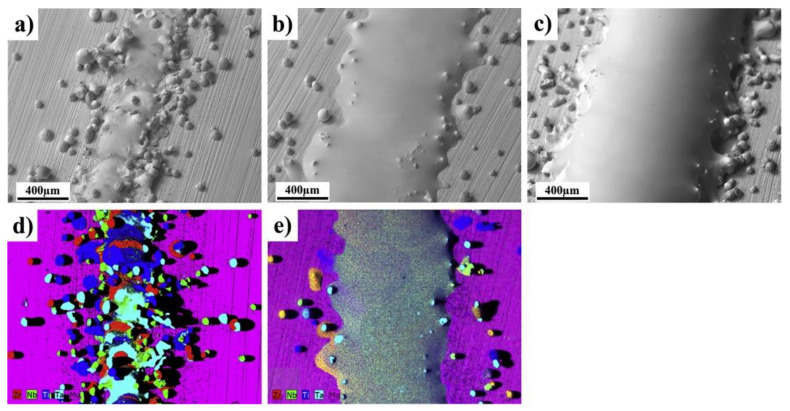
Deposition of TiZrNiTa refractory HEA on Mo substrate: (**a**) Single-track deposition; (**b**) Deposition after the second remelting step; (**c**) Deposition after four deposition and remelting steps; (**d**) The EDS mapping of deposition in (**a**) and (**e**) The EDS mapping of deposition in (b) [90]. (Reproduced with permission from Elsevier).

Therefore, apart from the flow properties of powder particles, the thermal interactions are also complex, which may affect the composition, microstructure, and performance. Metal elements cover a wide range of physical properties such as density, melting point, and laser absorption rate. For instance, among engineering structural alloys, the melting point can span from 600 °C in Al to 3400 °C in W [91]. The energy absorption rate is also worth considering when a highly reflected element, such as Cu, is part of the alloy [98]. In SLM work, a refractory HEA comprises of NbMoTaW was deposited [99]. A 3.5% composition deviation was found. Although the element composition of 5%–35% can all be regarded as HEA, how this deviation can affect the phase evolution and the final properties are still unknown at this moment. In [90], a new processing of DED was applied to deposit a functionally graded refractory HEA, which includes a wide range of melting points. The newly designed process route consists of a low power step to yield low powder evaporation and a high power step to remelt the previous layer and homogenize the elements. In Figure 9, Figure 9a,b are single track deposition and deposition track after the second remelting step, respectively. Figure 9d,e are EDS mapping of elements (Zr, Nb, Ti, Ta, and the substrate Mo) of Figure 9a,b respectively. From the EDS mapping, it can be seen that the remelting step strongly homogenizes the refractory elements within the entire deposition.

## 4. Outlook

Based on the major factors discussed in Section 3, it can be concluded that keeping an accurate composition in elemental powder deposition is still challenging. Those factors were partially studies in a couple of previous works. Each factor needs more experimental and theoretical studies. For blown powder-based DED, it was reported that linearly varying flow parameters can still result in nonlinear compositional grading and material behaviors [66]. The model of powder delivery using carrier gas needs to be further improved. Multiple factors, including the elemental powder properties and the processing parameters, are quite relevant, and the interactions among them are not negligible. It was reported that nano-sized powders were applied in high velocity oxygen fuel (HVOF) coating technique [100], which applies powder spray to fabricate thin layers. The delivery of powders with a more tiny size can be further studied that the DED process can be extended to more precise manufacturing applications. For thin layer deposition, preplacing powders on the processing surface before laser melting is another way to obtain a strong metallurgical bonding [50,51,52,53,54]. The dilution analysis can be further investigated to control the resultant phases after the deposition process.

There are very few works that cover the tolerance of chemical compositions of alloys fabricated by DED using elemental powders. Then, the compositional sensitivity study can be an important topic to study for the industry to adopt this processing method. It is worth mentioning that one advantage for elemental powder DED is that the mass loss due to evaporation can be compensated [36]. For pre-alloyed powders, since all the particles are in the same composition, if elements such as Mg, Zn, and Al are included, the favorable evaporation during the laser processing will change the overall composition. So, there is a need to compensate for this loss in pre-alloyed powders. However, for elemental powder mixture, more volatile elements can be prepared in the pre-designed powder mixture by adding more volatile element powders. Mukherjee et al. [101] listed some examples of the most volatile elements in some common alloys. Depositing pre-alloyed Ti-6Al-4V has a large loss in Al. It was reported the percentage of Al loss in pre-alloyed Ti-6Al-4V is higher than Cr loss in Inconel 625. If using elemental powders, various compositions can be pre-mixed to compensate for easy-to-evaporate alloy elements. An early compensation study was performed by Yan et al. [34]. During the first experiment, the pre-designed composition was equal to Ti-6Al-4V, the ideal value. However, the result gives Ti-5Al-2V. Later, a Ti-8Al-8V composition was pre-designed, and the result matched well with Ti-6Al-4V. More details for the compensation study in different alloy systems should be performed in future works, which is a challenging issue. A more mature relationship between the pre-mixed composition and the deposited composition should be studied. Another example is NiTi, which is very sensitive when there is a slight deviation in compositions. There is a need to figure out how to adjust the pre-designed composition in order to get an acceptable chemical composition.

Apart from obtaining an accurate composition, since the process is layer by layer, the heating and cooling history varies at different locations. Also, to build a bulk part, overlaps between tracks cannot be avoided. These are the main features in DED, and the grain structure and the anisotropic mechanical behavior resulted from the layer by layer heating and cooling history have been a hot topic. These features in DED lead to highly dynamic phenomena including dynamic melt pool, particle vaporization, rapid solidification and phase transition. In traditional manufacturing processes, highly dynamic phenomena was found to result in large scattering in mechanical properties [102], and when it comes to DED, the scattering can be more serious. When the feedstock materials become elemental powder mixture, the process will subject to more complex changes and may cause more discrepancies that have not been understood very well. The spatial difference sensitivity is worth learning, and a comprehensive thermophysical model is needed to control the temperature heating and cooling to guide the processing.

As a promising technology which uses elemental powders as feedstock materials, the effect of powder chemistry should be considered in the future study. Powder quality affects the final deposition, and for the DED process, little has known in this area. Powder chemistry, such as the composition of oxygen affects the final part of oxidation sensitive materials such as titanium alloys. Also, the oxidation of powder can induce porosity in the as-built parts. Karnati et al. [56] found the porosity issue in Cu-Ni FGM when using elemental Ni powder. A Ni alloy powder with 96 wt.% Ni and a small amount of Si was used as a substitution of pure Ni powder since Si can consume oxygen and relieve the porosity issue. Powder recycling is attracting research interest in recent years, which is also relevant to this area.

Last but not least, other related AM processing methods based on elemental powders, and the difference between elemental powder-based DED alloys and conventionally manufactured alloys are also worth further study. Nowadays, many important industrial alloys, such as Ti-6Al-4V and NiTi can be manufactured by both conventional methods and AM. The room-temperature mechanical properties of the as-fabricated Ti-6Al-4V using powder mixture were better than wrought counterparts [33]. In AM, selective laser melting (SLM) and electron beam melting (EBM) can also apply elemental powder mixtures. Wang et al. [49] found out the result of using SLM to fabricate NiTi alloy through elemental powders was not similar to DED. It was reported that there was a significant loss in Ti, which resulted in Ni-rich intermetallics as the predominant phase. The fabrication using EBM was not successful, which shows a low printability. Mechanisms and parametric study of SLM and EBM can be very different from DED based elemental powder manufacturing [49]. Studying in this aspect will give more understanding of the differences between additively manufactured alloys and conventionally manufactured alloys. It will also figure out more advantages of using multiple AM techniques to fabricate alloys by elemental powders in the corresponding industrial area.

## 5. Conclusions

In this paper, the current research status of using the DED process to fabricate metal alloys through elemental powder mixture was summarized based on different types of alloys, including industrial alloy and intermetallics synthesis, alloy modification, FGMs, metallic glass, and HEAs. The main issues and challenges are also summarized.

Various types of alloys, including industrial alloys, FGMs, metallic glass, and HEAs, have been synthesized by DED through elemental powders. Many of those works show good feasibility, and the mechanical properties of the deposited parts are comparable to conventionally manufactured alloys. As a new technique, numerous topics are still unsolved. Main scientific issues like overcoming the entropy of mixing, studying the physical and chemical properties of powders, the flow behavior for different metal powders, and how the laser-material interaction affect the final composition of the as-built part need systematic and in-depth research. Also, a relationship between the initial designed atomic or weight composition and the final composition is needed, and it will be integrated with the knowledge of materials science, dynamics, thermomechanical interaction, and the manufacturing system. Numerical simulation and more experimental results can be done in the future, which will significantly extend this new area to the metal manufacturing industry not only in DED, but also in other AM areas such as SLM and EBM.

## Figures and Tables

**Table 1:** Summary of Ti-based FGM alloys fabricated by elemental powder-based DED.

Alloy System	Ref.	Composition	Findings
Ti-Mo	[66]	Ti-25 at.% Mo	Hardness first increased and then decreased, a combination of grain size and alloy content.
Ti-Ta	[67]	Ti-50 wt.% Ta	The microhardness initially decreases, then increases, and finally decreases again.
Ti-Cr	[68]	Ti-60 at.% Cr	Hardness and modulus increase with Cr composition.
Ti-W	[70]	Ti-23 wt.% W	W has a significant effect of grain refinement across the gradient.

**Table 2:** Classification of alloys synthesized by elemental powder-based DED.

Alloy Types	Examples	Ref.
Industrial Alloys	Ti-6Al-4V	[33,34,35,36]
	Stainless Steel	[37]
Intermetallics	FeAl	[39]
	NiTi	[44,45,46,47,48,49]
	Ni_3_Al	[50]
	Hard Coadings: TiCo, Cr_3_Si, NiSi, etc.	[51,52,53,54,55]
FGMs	Ti-based: Ti-Mo, Ti-V, Ti-Ta, etc.	[68,69,70,71,72]
	Cu-Ni	[73]
	Fe-Cr-Ni	[74,75]
Magnetic Alloys	Fe73.5Si13.5B9Nb3Cu1 etc.	[76,77]
Metallic Glass	78Fe10BC9Si2Al1C etc.	[78,79]
HEAs	Al_x_CrCuFeNi etc.	[81,82,83,84,85,86,87,88,89,90,91]
